# Supplementary material for: Inequality in Health: The Correlation between Poverty and Injury—A Comprehensive Analysis Based on Income Level in Taiwan: A Cross-Sectional Study
Source: Healthcare (Basel). 2021 Mar 18;9(3):349. doi: 10.3390/healthcare9030349 (PMC8003303; doi:10.3390/healthcare9030349)
Supplement: Supplementary file 1 [file healthcare-09-00349-s001.pdf]

Table 1. ISS (Injury severity score) distribution of injury inpatients for various incomes during 1998-2015 in Taiwan (n = 4,647,058).

Prognosis	Overall					Non-fatal (Survival)					Fatal (Mortality)				
Incomes	Low-income (n = 74,337)		Non-low-income (n = 4,572,721)		P value	Low-income (n = 71,640)		Non-low-income (n = 4,477,412)		P value	Low-income (n = 2,697)		Non-low-income (n = 95,309)		P value
ISS score	N	%	N	%		N	%	N	%		N	%	N	%	
<16	60,826	81.8	4,100,676	89.7	<0.001	58,816	82.1	4,025,191	89.9	<0.001	2,010	74.5	75,485	79.2	<0.001
≧16	13,511	18.2	472,045	10.3	<0.001	12,824	17.9	452,221	10.1	<0.001	687	25.5	19,824	20.8	<0.001

P value: percentage test

Table S2. Age distribution of fall injury inpatients for various incomes during 1998-2015 in Taiwan (n =726,651)

Prognosis	Overall					Non-fatal (Survival)					Fatal (Mortality)				
Incomes	Low-income (n = 12,184)		Non-low-income (n = 714,467)		P value	Low-income (n = 11,810)		Non-low-income (n = 701,304)		P value	Low-income (n = 374)		Non-low-income (n = 13,163)		P value
Age group (years)	N	%	N	%		N	%	N	%		N	%	N	%	
1-4	176	1.4	22,200	3.1	0.194	176	1.5	22,050	3.1	0.222	0	0	150	1.1	<0.001
5-14	1,304	10.7	53,223	7.4	<0.001	1,304	11.0	53,183	7.6	<0.001	0	0	40	0.3	<0.001
15-24	550	4.5	33,573	4.7	0.826	548	4.6	33,401	4.8	0.828	2	0.5	172	1.3	0.921
25-44	1,651	13.6	106,444	14.9	0.141	1,623	13.7	105,287	15.0	0.146	28	7.5	1,157	8.8	0.810
45-64	2,958	24.3	167,303	23.4	0.252	2,857	24.2	164,943	23.5	0.382	101	27.0	2,360	17.9	0.021
≧65	5,545	45.5	331,724	46.4	0.139	5,302	44.9	322,440	46.0	0.111	243	65.0	9,284	70.5	0.064

P value: percentage test

Table S3. The injuried of traffic injury inpatients for various incomes during 1998-2015 in Taiwan (*n* = 1,175,912)

Prognosis	Overall					Non-fatal (Survival)					Fatal (Mortality)				
Incomes	Low-income (*n* =13,723)		Non-low-income (*n* =1,162,189)		*P* value	Low-income (*n* = 13,417)		Non-low-income (*n* = 1,146,981)		*P* value	Low-income (*n* = 306)		Non-low-income (*n* = 15,208)		*P* value
**The injuried**	N	%	N	%		N	%	N	%		N	%	N	%	
Driver of motor vehicle	1,835	13.4	287,701	24.8	<0.001	1,784	13.3	284,113	24.8	<0.001	51	16.7	3,588	23.6	0.032
Passenger in motor vehicle	174	1.3	15,264	1.3	0.987	173	1.3	14,912	1.3	0.992	1	0.3	352	2.3	0.432
Motorcyclist	9,386	68.4	727,170	62.6	<0.001	9,244	68.9	719,154	62.7	<0.001	142	46.4	8,016	52.7	0.001
Passenger on motorcycle	1,021	7.4	41,758	3.6	<0.001	1,018	7.6	41,290	3.6	<0.001	3	1.0	468	3.1	0.295
Pedal cyclist	514	3.7	34,235	2.9	0.245	467	3.5	33,262	2.9	0.520	47	15.4	973	6.4	<0.001
Pedestrian	730	5.3	54,548	4.7	0.720	682	5.1	52,768	4.6	0.608	48	15.7	1,780	11.7	0.004
Others	63	0.5	1,513	0.1	0.138	49	0.4	1,482	0.1	0.614	14	4.6	31	0.2	0.088

P value: percentage test

Driver of motor vehicle: ICD-9-CM E800.x0-E849.x0, Passenger in motor vehicle: ICD-9-CM E800.x1-E849.x1; Motorcyclist: ICD-9-CM E800.x2-E849.x2; Passenger on motorcycle: ICD-9-CM E800.x3-E849.x3; Pedal cyclist: ICD-9-CM E800.x6-E849.x6; Pedestrian: ICD-9-CM E810.x7-E819.x7; Others: ICD-9-CM E800-E849, excluding above
